# Editorial to the Special Issue on Skin Cancer: The State of the Art

**DOI:** 10.3390/ijms23073806

**Published:** 2022-03-30

**Authors:** Piotr Rutkowski, Andrzej Mackiewicz

**Affiliations:** 1 Department of Soft Tissue/Bone Sarcoma and Melanoma, Maria Skłodowska-Curie National Research Institute of Oncology, 02-781 Warsaw, Poland; 2 Medical Biotechnology, Poznan University of Medical Sciences, 61-866 Poznan, Poland; 3 Department of Cancer Diagnostics and Immunology, Greater Poland Cancer Centre, 61-866 Poznan, Poland

This Special Issue of the *International Journal of Molecular Sciences* focuses on skin cancers, specifically on the rare forms of these tumors. Here, we present a series of manuscripts prepared by multidisciplinary experts that are valuable for diagnosis and therapy in routine practice. 

Skin carcinomas, also defined as non-melanoma skins cancers (NMSC), are malignancies with a marked preference for lighter-skinned people and are responsible for about 1/3 of all new cancer diagnosed in humans [1]. Despite a relatively low death risk and low metastatic potential, they remain a significant clinical challenge because they tend to infiltrate surrounding structures and may be characterized by a high propensity for local recurrences. In high-risk patients, the course of the disease may be different because skin carcinomas in these patients are more aggressive and often result in death. The same issue also exists for rare, aggressive types of skin carcinomas, such as Merkel cell carcinoma.

On the other hand, melanoma accounts for a low percentage of all skin malignancies but is responsible for most deaths related to cutaneous cancers [2].

Understanding the mechanisms of the etiopathogenesis of skin cancers resulted in the development of new treatment modalities that revolutionized the therapy of advanced melanoma and cutaneous carcinomas. The survival of advanced, unresectable metastatic melanoma and skin carcinomas has been greatly improved within the last few years due to progress in immunotherapy (mostly related to immune checkpoint inhibitors anti-PD-1/PD-L1 and anti-CTLA-4) and targeted therapy (BRAF and MEK inhibitors in melanoma and hedgehog inhibitors in basal cell carcinoma) [3,4].

In metastatic melanoma, the current results of anti-PD-1 therapy (monotherapy with pembrolizumab or nivolumab) show a median overall survival (OS) of approximately 2 years and 5-year OS rates at the level of 40%, but combined immunotherapy with anti-PD-1 and anti-CTLA-4 (nivolumab with ipilimumab) is superior in terms of progression-free and OS, with recent data confirming metastatic melanoma median OS of more than 5 years, for the first time ever (72.1 months based on the long-term results of the CheckMate 067 trial) [5]. However, some rare melanoma subtypes, such as mucosal melanoma, may lower the efficacy of immunotherapy [6]. Indini et al. prepared a comprehensive review on novel therapeutic strategies and molecular profiling in mucosal melanoma [7]. Mucosal melanoma harbors distinct molecular features from skin melanoma characterized by the ultraviolet gene signature. Mucosal melanoma usually has a lower mutational tumor burden, with fewer nucleotide substitutions per cell, but more gene amplifications than cutaneous melanoma. Moreover, in mucosal melanoma, v-Raf murine sarcoma viral oncogene homolog B (*BRAF*) gene mutations are found less commonly, but a higher incidence of tyrosine-protein kinase KIT (CD117) oncogene mutations is pronounced [8]. In a recent study by our research group [9], we found a median OS of 16.3 months in a whole cohort of patients with advanced melanoma treated with anti-PD-1 immunotherapy, which is lower than that reported in contemporary groups of patients with cutaneous melanoma. Patients who received radiotherapy during anti-PD1 treatment had a better median PFS of 8.9 months than patients only treated with anti-PD1 monotherapy (median PFS of 4.2 months). We can conclude, similarly to other series, that anti-PD1 antibodies are an effective treatment option in advanced mucosal melanoma patients but with poorer outcomes than cutaneous melanoma. These results also implied that the addition of radiotherapy might be beneficial in a selected subgroup of patients with mucosal melanoma. Recent results from pooled analyses of patients with mucosal melanoma treated with immunotherapy suggested that the combination of immune checkpoint inhibitors may improve survival in these patients compared with single-agent immunotherapy. The presence of *KIT* mutations constitutes potential targets of tyrosine kinase inhibitors currently in use in the clinic (as imatinib or nilotinib) [10].

The second paper in this Special Issue concerns Merkel cell carcinoma (MCC), which is a very rare type of skin carcinoma, making its diagnosis challenging. In this review, Stachyra et al. [11] widely review diagnostics hints, the molecular background, and new therapeutic strategies. MCC is highly immunogenic due to pathogenesis related to ultraviolet radiation and Merkel cell polyomavirus (MCPyV) infection. The treatment of advanced MCC was revolutionized by introducing immune checkpoint inhibitors, such as avelumab (anti-PD-L1) and pembrolizumab (anti-PD-1). The efficacy of avelumab in the treatment of MCC has been proven in the JAVELIN Merkel 200 phase-II, single-arm clinical trial. The median PFS (progression-free survival) was 2.7 months (in the second line of treatment), and the percentage of patients free from disease progression after 6 months was 40%. The PFS reached a plateau. The OS rate after 6 months was 69%, the median OS was 11.3 months, and the overall response rate (ORR) was 33% [12]. The updated results of part B of this trial confirmed that 77.8% (14 out of 18) of treatment responses were maintained, and the response duration in 83% of cases was longer than 6 months [13]. Pembrolizumab was only approved in the US based on KEYNOTE-017 first-line trial results. The ORR was 56%; in 28 patients with responses, 54% had a response duration of more than 12 months. The PFS rate after 24 months was 48.3%, with a median PFS of 16.8 months; the OS rate after 24 months was 68.7%, and the median OS was not reached [14,15]. 

The last two papers in this Special Issue focus on current approaches in extremely rare skin carcinomas—cutaneous adnexal neoplasms. Płachta et al. [16,17] summarized the contemporary approach to cutaneous adnexal neoplasms with apocrine, eccrine, and follicular differentiation. We would like to congratulate the authors’ wide review of available knowledge in these groups of rare tumors, which makes these two manuscripts extremely useful in routine clinical practice.
